# Titer- and Intervention Timing-Dependent Functional Effects of AAV9-NeuroD1 Gene Therapy on Spinal Cord Injury

**DOI:** 10.3390/cimb48080786

**Published:** 2026-08-02

**Authors:** Alex Roman, Maggie Sorensen, Ezequiel Marron Fernandez de Velasco, Ann M. Parr, Andrew W. Grande, Walter C. Low

**Affiliations:** 1 Graduate Program in Neuroscience, University of Minnesota, Minneapolis, MN 55455, USA; 2 Department of Neurosurgery, University of Minnesota, Minneapolis, MN 55455, USA; 3 College of Biological Sciences, University of Minnesota, Minneapolis, MN 55455, USA; 4 Viral Vector and Cloning Core, University of Minnesota, Minneapolis, MN 55455, USA; marro014@umn.edu

**Keywords:** NeuroD1, AAV9, spinal cord injury, in vivo reprogramming, astrocyte, neuroprotection, regenerative medicine

## Abstract

Spinal cord injury (SCI) often results in varying degrees of motor and sensory dysfunction with limited potential for recovery. Research into in vivo astrocyte-to-neuron reprogramming has led to promising results that, if translated to SCI, could offer substantial therapeutic benefit by replenishing lost populations of neurons for functional restoration. Previous studies have shown that AAV9-mediated delivery of NeuroD1 is capable of reprogramming astrocytes into neurons in vivo after chronic SCI in rats. Here we evaluate the dose-dependent functional and neuroprotective potential of the NeuroD1 gene therapy platform for acute and subacute SCI in rats. The Cre-dependent, DIO-based AAV9-NeuroD1 gene therapy platform was administered directly into the spinal cord of female Long-Evans rats after moderate, thoracic level 8/9 contusion SCI at one of two intervention timepoints: immediately after injury (acute) or 1 week post-contusion (subacute). The viruses were administered at a titer of 10^11^ or 10^13^ GC/mL. We demonstrate that the AAV9-NeuroD1 reprogramming platform successfully, albeit variably, transduced spinal cells that persisted up to 6 weeks post-injury. However, histological analysis revealed substantial neuronal off-targeting, suggesting a lack of astrocyte-specific targeting from the AAV9 DIO-based delivery platform. Surprisingly, we also found titer- and intervention timing-dependent effects on motor function and tissue preservation—as demonstrated by 1–2 point decrease in BBB scoring and near 50% increase in peak lesion cavitation area—when AAV9-NeuroD1 was administered immediately after SCI. While did not find any effects of the AAV9-NeuroD1 platform on sensory function or neuroinflammatory cell density, a subset of NeuroD1 signal reflected phagocytic uptake by Iba1 and CD68-expressing microglia/macrophages. These results indicate that the NeuroD1 reprogramming platform can exacerbate injury-related functional deficits when administered in the acute stage of injury, but has neuroprotective benefit on tissue preservation when administered in the subacute stage of injury. Our study demonstrates that several factors must be considered, including viral titer and intervention timing, to assess the therapeutic potential of AAV9-NeuroD1-mediated reprogramming for SCI.

## 1. Introduction

Traumatic spinal cord injury (SCI) is a globally prevalent injury caused by damage to the spinal cord, the vertebrae, or the surrounding spinal nerves that often result in irreversible consequences for the central nervous system (CNS). Depending on the severity and location of the injury, it can lead to varying degrees of motor and sensory dysfunction classified by the completeness of the resulting paraplegia or quadriplegia [1]. Additionally, on a molecular level, the injured spinal cord is characterized by a myriad of pathophysiological processes including neuronal cell death [1,2,3], demyelination [1,4], reactive gliosis [1,2,5], neuroinflammation [1,2], lesion cavity formation [1,2,6], and the development of a glial scar [1,2,7]. These cascading consequences of the secondary injury create a complex injury environment that extends well into the subacute and chronic stages of injury, often acting as barriers to attempts at experimental therapeutic intervention. Consequently, current SCI research is focused on developing a therapeutic approach that addresses components of the complex injury site with the goal of restoring nervous system function after injury.

Reactive astrocytes are integral to secondary injury progression due to their multifaceted and complex role throughout the injury response. Resident astrocytes are a population of glial cells within the CNS responsible for synaptic transmission modulation [8,9] and metabolic regulation [10]. After traumatic CNS injury, astrocytes become reactive, upregulate GFAP expression [11], and change their morphology and function [7,12,13]. The reactive astrocyte population—boosted by proliferation and recruitment to the injury site [1,5,7,12]—is involved in reactive gliosis and acute neuroinflammation [12,14,15]. From the subacute stage extending to the chronic stage of injury, reactive astrocytes express chondroitin sulfate proteoglycans—potent inhibitors of axonal growth [12,13]—and are integral to forming and maintaining the glial scar [1,2,5,12]. As a result of their impact on injury progression, reactive astrocytes have been promising targets for therapeutic intervention.

In vivo astrocyte-to-neuron reprogramming is an emerging field of therapeutic intervention that targets endogenous reactive astrocytes with overexpression of select neurogenic transcription factors for transdifferentiation into functional neurons in animal models of CNS injury and disease [16]. Across these studies, several transcription factors have been identified as being capable of converting astrocytes into neurons, including NeuroD1 [17,18,19,20,21], Ngn2 [22,23], Dlx2 [20,24], and Ascl1 [20,25]. While the majority of astrocyte-to-neuron reprogramming studies using these induction factors have been evaluated for brain injuries and diseases, recent studies have also assessed the reprogramming platform within the context of animal models of SCI. In particular, adeno-associated virus (AAV)-mediated expression of NeuroD1 has been shown capable of generating functional glutamatergic neurons in both the brain [21] and the spinal cord [17]. Of interest, in a mouse model of traumatic brain injury, NeuroD1 was demonstrated to reprogram glial scar-forming astrocytes into neurons [26]. Considering that the glial scar, a prominent feature of human SCI, is often cited as a barrier to axonal growth and circuit regeneration [27], targeting glial scar-forming reactive astrocytes for transdifferentiation into neurons is an appealing strategy for SCI therapeutic intervention. Further, NeuroD1 has also been shown to decrease neuroinflammation when expressed in striatal astrocytes alongside Dlx2 [24], potentially offering additional neuroprotective benefit.

While NeuroD1-mediated astrocyte-to-neuron reprogramming appears to be a promising approach to functional restoration after SCI, the reprogramming platform has been met with skepticism. Despite the studies claiming that in vivo single-factor conversion of astrocyte into neurons is possible, other studies were unable to produce similar results, instead finding evidence to suggest that endogenous neurons, rather than reactive astrocytes, were transduced by the viral reprogramming platform [28,29]. These researchers hypothesized that previously reported benefits attributed to NeuroD1 treatment may have instead been facilitated by a neuroprotective effect, not reprogramming [28], thus highlighting the complexity and potential inefficiency of the reprogramming paradigm when translating to different in vivo models of injury and disease. In response, additional studies have begun to assess key factors that may influence AAV-based NeuroD1-mediated reprogramming efficacy. One such factor is viral titer, where ‘high’ titers of AAV for NeuroD1 transduction have, in part, contributed to the observed neuronal off-targeting [30]. Furthermore, the intervention timing of NeuroD1 treatment for SCI has yet to be fully explored seeing as the platform has yet to be evaluated as an experimental therapeutic approach for acute SCI, a neurobiologically delicate and complex stage of injury that may be benefitted by the reprogramming—and potentially neuroprotective—treatment platform. Ultimately, additional research is needed to fully characterize the efficiency and nuances of the NeuroD1-mediated astrocyte-to-neuron reprogramming platform as well as its effects on functional outcomes, particularly for SCI.

This study seeks to evaluate the AAV9-NeuroD1-based reprogramming platform for acute and subacute SCI, exploring its dose-dependent potential for motor and sensory functional improvement as well as its potential neuroprotective effects. Consistent with a previous study that evaluated NeuroD1 reprogramming for chronic SCI [17], we used a Cre-dependent AAV9 delivery platform with a GFAP promoter for selective targeting of spinal cord reactive astrocytes with NeuroD1 expression in a rat model of moderate contusion SCI. We assessed titer-dependent motor and sensory functional recovery in response to the AAV9-NeuroD1 reprogramming for 6 weeks post-contusion (WPC). To characterize the potential neuroprotective effects, we measured tissue and lesion area across the injury site as well as levels of neuroinflammation. In response to acute AAV9-NeuroD1 viral administration, our study revealed a surprising titer-dependent deleterious effect on motor functional recovery, lesion cavity size, and spared tissue at 6 WPC. This is in contrast to subacute viral administration, where NeuroD1 treatment had limited to no effect on functional or neuroprotective outcomes. However, regardless of intervention timing, exploratory histological analyses revealed titer-independent neuronal off-targeting from the Cre-dependent, AAV9-based DIO delivery platform.

## 2. Materials and Methods

### 2.1. Spinal Cord Contusion Injury

This study used adult female Long-Evans rats weighing 220–250 g (Charles River Laboratories, Wilmington, MA, USA). Female rats were chosen for consistency within the field of SCI modeling [31] and to avoid male-specific severe post-operative complications [31,32]. All procedures were approved by the Institutional Animal Care and Use Committee at the University of Minnesota Twin Cities. A total of 2 h prior to surgery, extended-release buprenorphine [0.65 mg/kg] was administered subcutaneously. Rats were anesthetized with isoflurane inhalation then a T8/T9 laminectomy was performed. A moderate contusion injury was performed with the Infinite Horizon Impactor (IH-0400; Precision Systems and Instrumentation, Lexington, KY, USA) programmed to administer a 200 kDyn force at the T8/T9 vertebral level (Figure S1). Following the surgery, the injury site was closed in layers. The rats were then placed on heating pads and closely monitored for recovery. Ceftiofur [10 mg/kg] was administered subcutaneously for 5 days post-operatively to prevent infection. Additionally, considering the level and severity of injury, the bladders were manually expressed beginning with twice daily and continuing as needed.

### 2.2. Acute and Subacute Intraspinal Viral Injection

All viruses were produced at the University of Minnesota Viral Vector and Cloning Core. This study makes use of AAV9 vectors—hGFAP::Cre, CAG::DIO-mRuby2, CAG::DIO-NeuroD1-T2A-mRuby2—in a two-vector AAV9 DIO delivery platform designed to selectively drive persistent mRuby2 or NeuroD1-mRuby2 expression in GFAP-expressing cells. Throughout this study, the control treatment refers to the simultaneous administration of hGFAP::Cre and CAG::DIO-mRuby2 whereas the NeuroD1 treatment refers to the simultaneous administration of hGFAP::Cre and CAG::DIO-NeuroD1-T2A-mRuby2. Virus titers, as measured by genome copies/mL (GC/mL), were 2.4 × 10^13^ GC/mL for hGFAP::Cre, 2.5 × 10^13^ GC/mL for CAG::DIO-mRuby2, and 2.5 × 10^13^ GC/mL for CAG::DIO-NeuroD1-T2A-mRuby2. This study uses the viruses at two different titers, ‘high’ and ‘low’ titer, where the high titer is the starting titer as described and the low titer is a 1:100 dilution of the high titer. This resulted in four acute experimental groups—SCI-only (*n* = 7), control [high] (*n* = 7), NeuroD1 [low] (*n* = 7), and NeuroD1 [high] (*n* = 9)—and five subacute experimental groups—SCI-only (*n* = 9), control [low] (*n* = 11), control [high] (*n* = 8), NeuroD1 [low] (*n* = 11), and NeuroD1 [high] (*n* = 10).

All rats received a moderate thoracic SCI as described. For acute administration, rats received one of the following treatments immediately after contusion injury and prior to injury site closure: control [high], NeuroD1 [low], NeuroD1 [high], or no treatment (SCI-only). For rats that received a viral treatment, they were placed under a stereotaxic frame immediately after contusion injury. Using the Quintessential Stereotaxic Injector (Product No. 53313; Stoelting Co., Ltd., Wood Dale, IL, USA), the needle of a 10 µL Hamilton syringe containing 3 µL of virus—1.5 µL of hGFAP::Cre and 1.5 µL of either CAG::DIO-mRuby2 or CAG::DIO-NeuroD1-T2A-mRuby2, depending on treatment—was situated above the lesion center. At an injection rate of 0.5 µL/min, 1.5 µL of the combined viruses were administered in two locations, 1.0 mm rostral and 1.0 mm caudal to the injury site, both at a depth of 2.0 mm. To prevent reflux, the needle was removed 5 min after administering the virus. After intraspinal viral injection, the injury site was closed in layers. For subacute administration, the rats were prepared for surgery at 1 WPC following the same preparatory procedures used for the SCI surgery. The injury site was then re-exposed, and the viruses were administered for the following treatments as previously described: control [low], control [high], NeuroD1 [low], or NeuroD1 [high]. Rats that did not receive treatment (SCI-only) did not undergo this second surgery.

The treatment type for each animal was randomly determined and accounted for timing and order throughout each surgical session; equal numbers of animals in each treatment group underwent the surgical procedure at each position throughout all surgical sessions (e.g., first, second, third, etc.). This was done to minimize the potential confounding effect of the order of administered treatments.

### 2.3. Behavioral Testing

For motor testing, rats were assessed using Basso, Beattie, and Bresnahan (BBB) open field locomotor testing [33]. The rats were evaluated the day before SCI as a baseline and weekly thereafter up to 6 WPC. Researchers were blinded to the treatment group during testing.

For somatosensory testing, rats were assessed using the von Frey filament test for mechanical sensitivity and the cotton swab test for light touch, dynamic sensation. For both somatosensory tests, the rats were habituated on an elevated platform with a perforated metal sheet flooring for 1 h prior to testing. The von Frey filament test was performed using the Up–Down technique to measure 50% paw withdrawal threshold for both hindpaws and the left forepaw as a control [34]. The cotton swab test was performed using a cotton-tipped applicator that was “puffed out” to approximately three times its original size. The cotton swab was then brushed along the paw in the heel-to-toe direction, where the frequency of responses was observed. These trials were repeated 10 times for each paw, alternating between both hindpaws and the left forepaw, with an interval of 1 min between each test [35]. The rats were evaluated the day before SCI as a baseline and weekly thereafter up to 6 WPC. Researchers were blinded to the treatment group during testing.

### 2.4. Spinal Cord Tissue Harvesting

At 6 WPC, after the final behavioral testing was completed, rats were anesthetized with isoflurane inhalation then transcardially perfused with 4% paraformaldehyde in 0.01 M PBS. The spinal cords were then extracted and post-fixed overnight in the same 4% paraformaldehyde solution at 4 °C. The spinal cords were then immersed in 10% (*w*/*v*) sucrose in PBS until the tissue sank. The tissue was then immersed in 20% (*w*/*v*) sucrose, followed by 30% (*w*/*v*) sucrose. A 1.8 cm segment of the spinal cord containing and centered around the lesion epicenter was then removed and embedded in clear Tissue Plus OCT Compound (Fisher HealthCare, Waltham, MA, USA). The tissue was then cryosectioned in the transverse plane at 20 µm thickness using the Leica JUNGCM1800 cryostat.

### 2.5. Histology

To analyze the lesion cavity and spared tissue, cryosectioned tissue from all experimental groups were stained with hematoxylin and eosin (H&E) (H&E Staining Kit, ab245880; Abcam, Waltham, MA, USA). Slides containing the tissue sections of interest were hydrated from 100% ethanol to 95% ethanol. The sections were rinsed with distilled water then incubated in Modified Mayer’s Hematoxylin for 5 min. The sections were then rinsed under running tap water for 3 min. After a 15 s incubation in Bluing Reagent, the sections were rinsed with distilled water, dipped in 100% ethanol, then incubated in Eosin Y (Modified Alcoholic). The sections were then dehydrated from 95% ethanol to 100% ethanol, cleared with xylene, and coverslipped with Eukitt Quick-Hardening Mounting Media (Sigma-Aldrich, St. Louis, MO, USA).

To assess the extent of successful transduction and characterize the identity of virally transduced cells, cryosectioned tissue from all experimental groups was processed for immunohistochemistry (IHC) staining and analysis. In brief, following a 0.01 M PBS wash and 0.1% PBST permeabilization, sections were blocked for 1 h, at room temperature, with a PBS solution containing 5% normal horse serum. The sections were then incubated overnight at 4 °C with primary antibodies in an incubation solution containing 0.01 M PBS, 0.3% triton X-100, 1% bovine serum albumin, and 2% normal donkey serum. Sections were then washed twice with PBS, permeabilized with 0.1% PBST, and incubated with the corresponding secondary antibodies conjugated with Alexa Fluor 488 and Alexa Fluor 647 for 1 h at room temperature. After PBS washes, sections were counterstained with DAPI, rinsed with PBS, then coverslipped with Immu-Mount mounting medium (Fisher Scientific, Waltham, MA, USA). The primary antibodies used were chicken anti-GFAP (1:1000, LifeSpan Biosciences, Newark, CA, USA), mouse anti-NeuN (1:500, Abcam, Waltham, MA, USA), rabbit anti-Iba1 (1:500, FUJIFILM Wako, Richmond, VA, USA), and mouse anti-Rat CD68 (1:200; Bio-Rad Laboratories, Hercules, CA, USA). The following secondary antibodies were conjugated with Alexa Fluor 488: donkey anti-chicken (1:1000; Abcam, Waltham, MA, USA) and donkey anti-mouse, 1:1000; Abcam, Waltham, MA, USA). The following secondary antibodies were conjugated with Alexa Fluor 647: donkey anti-rabbit (1:1000; Abcam, Waltham, MA, USA) and donkey anti-mouse (1:1000; Abcam, Waltham, MA, USA).

### 2.6. Imaging and Quantitative Analyses

For H&E-stained tissue, select sections representing the spinal cord at the lesion epicenter and every 500 µm, up to 5 mm in both the rostral and caudal directions, were imaged on a Leica DMi8 inverted microscope. The total tissue area and corresponding lesion area were then traced and measured using Fiji ImageJ software (v.1.54f, National Institutes of Health, Bethesda, MD, USA).

For IHC-stained tissue, select sections representing the spinal cord were imaged on a Nikon AX R confocal microscope. Images of full spinal cord sections were acquired with resonant scanning using a 20× objective. Depending on the size of the section, the large image was acquired by a 3 × 3 or 4 × 4 scan area, with a 7-step symmetrical z-stack at a range of 10 µm. The resulting multipoint images were then denoised and stitched together using blending with shading correction. Finally, extended depth of focus (EDF) was used to generate the 2D projection image from the z-stack images. To obtain images for mRuby2 characterization, a 20× objective was used at a zoom size of 3.0× for appropriate spatial resolution at 4 particular locations: gray commissure, ventral horn, ventral funiculus, and lateral funiculus. For each sampling location, the image was acquired with a z-stack at a range of 15 µm and a step size of 0.3 µm. The resulting image was then 3D deconvoluted, denoised, and EDF was used to generate the final 2D image for quantification. Fluorescent co-labeling between mRuby2 and targeted proteins—GFAP, NeuN, Iba1, and CD68—at 3.0 mm rostral and caudal to the injury site were assessed with QuPath. For neuroinflammatory marker (Iba1 and CD68) density, images of the spinal cord—at 3.0 mm rostral and caudal to the injury site—were taken on a Leica Dmi8 inverted microscope at 10×. The resulting images were then analyzed for neuroinflammatory marker density in QuPath at the same 4 locations as the mRuby2 co-labeling analysis.

### 2.7. Statistical Analysis

All data are described and visualized as mean ± standard error. BBB scores, von Frey 50% paw withdrawal thresholds, and cotton swab paw withdrawal frequencies were analyzed using repeated measures two-way analysis of variance (ANOVA). For behavioral data analysis, animals were excluded if their baseline was 2 SD away from the mean. Exclusion of these subjects had no effect on statistical outcomes. Lesion cavity analysis and quantitative IHC data were analyzed using two-way ANOVA. Tukey’s post hoc test was used to identify significant differences among treatment groups, where significance was determined by *p* < 0.05.

## 3. Results

### 3.1. Acute AAV9-NeuroD1 Successfully Transduces Cells in the Injured Spinal Cord

**Figure 1:**
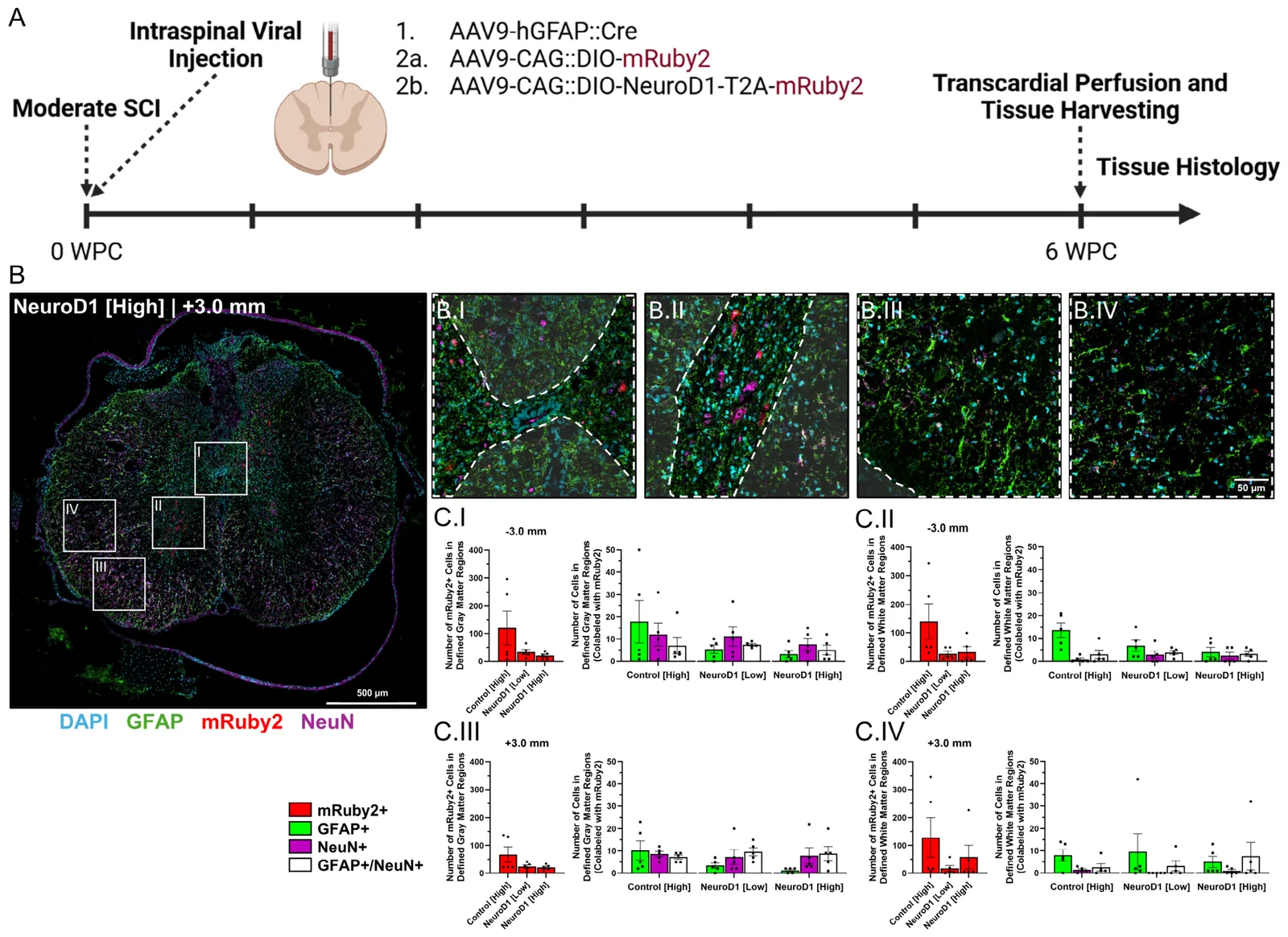
NeuroD1 transduction in the spinal cord after acute intervention. (**A**) Rats received a moderate thoracic SCI which was followed immediately by intraspinal viral injection of AAV9 viruses, depending on treatment groups. control-treated rats were injected with hGFAP::Cre and CAG::DIO-mRuby2, whereas NeuroD1-treated rats were injected with hGFAP::Cre and CAG::DIO-NeuroD1-T2A-mRuby2; SCI-only rats did not receive any viral injection. The rats were perfused at 6 WPC, and spinal cords were extracted and prepared for tissue histology. (**B**) IHC was performed on spinal cord tissue, staining for GFAP (green), NeuN (magenta), and counterstained with DAPI (cyan); mRuby2, the reporter protein for successful viral transduction, is shown in red. A representative spinal cord section from a NeuroD1 [high]-treated spinal cord at 3.0 mm caudal to the injury site is also shown. mRuby2 cell quantification and co-labeling analyses were then performed at 4 select locations for each spinal cord section: (**B.I**) central canal, (**B.II**) ventral horn, (**B.III**) ventromedial white matter, and (**B.IV**) ventrolateral white matter. These regions are approximated on the full representative spinal cord image by white boxes. (**C.I**–**C.IV**) Quantification of total mRuby2+ cells and their co-labeling with GFAP, NeuN, or both GFAP and NeuN in spinal cord sections at 3.0 mm rostral (−3.0 mm; (**C.I**,**C.II**)) or 3.0 mm caudal (+3.0 mm, **C.III**,**C.IV**) from the injury site (*n* = 5 for each experimental group). Defined gray matter and white matter regions are represented by combining the areas within the white dashed lines in (**B.I**,**B.II**) and (**B.III**,**B.IV**), respectively. All data shown are presented as mean ± standard error.

We aimed to investigate the efficiency of the AAV9-based, NeuroD1-mediated astrocyte-to-neuron reprogramming platform when administered in the acute stage of moderate contusion SCI in rats. To this end, we used a two-vector, DIO Cre-based approach for select NeuroD1 expression in reactive astrocytes, as previous reprogramming studies have demonstrated successful transduction with this method [17,36,37]. This AAV9-NeuroD1 reprogramming platform makes use of two AAV9 vectors that are administered simultaneously. The first virus, AAV9-hGFAP::Cre, contains Cre recombinase under the control of a human GFAP promoter, such that only GFAP-expressing cells, namely reactive astrocytes, will express the Cre recombinase protein. The second virus contains a Cre-dependent cassette for the expression of the mRuby2 reporter protein alone (AAV9-CAG::DIO-mRuby2; control treatment), or NeuroD1 with mRuby2 (AAV9-CAG::DIO-NeuroD1-T2A-mRuby2; NeuroD1 treatment) under a ubiquitous CAG promoter (Figure 1A). This is such that NeuroD1-mRuby2 or mRuby2 alone will be expressed only in cells expressing the Cre recombinase protein; since the NeuroD1 and mRuby2 sequences are under a CAG promoter, their expression will continue after the intended astrocyte-to-neuron conversion. The platform specifically targets GFAP-expressing reactive astrocytes with Cre recombinase expression to allow for translation and persistent expression of the NeuroD1-mRuby2, resulting in select astrocyte-to-neuron reprogramming.

We used this reprogramming platform in a rat model of moderate contusion SCI at the thoracic 8/9 (T8/T9) level. As an acute treatment, we injected the viruses immediately after the spinal cord was injured. The viruses were administered directly into the spinal cord at 1.0 mm rostral and 1.0 mm caudal to the injury site. Considering the study that identified the role that viral titer may play in transduction selectivity [38], we also sought to assess the NeuroD1 treatment at two viral titers; the NeuroD1 viral treatment was administered at a viral titer of either 10^13^ GC/mL or 10^11^ GC/mL, or NeuroD1 [high] and NeuroD1 [low], respectively. The control treatment was administered at the high-titer-only—control [high]. At 6 WPC, the rats were perfused, and the spinal cords were extracted and prepared for histological analysis (Figure 1A).

To assess the efficacy of viral transduction, we looked at the expression of mRuby2 and how it co-labeled with antibody markers for reactive astrocytes (GFAP) and neurons (NeuN) (Figure 1B). We stained sections of the spinal cord at 3.0 mm rostral (−3.0 mm) and 3.0 mm caudal (+3.0 mm) to the site of injury; these distances from lesion center were chosen for the amount of intact, non-lesioned tissue and distance from the injection site. For each spinal cord section imaged, four locations were assessed based on trends in abundant mRuby2 expression: I. central canal gray matter, II. ventral horn gray matter, III. ventromedial white matter, and IV. ventrolateral white matter. The number of mRuby2+ cells was measured within the gray matter regions (Figure 1B.I,B.II) and white matter regions (Figure 1B.III,B.IV) of the assessed images (*n* = 5 for each experimental group). The identities of the mRuby2+ cells were determined by way of co-labeling with GFAP alone, NeuN alone, or both GFAP and NeuN within the defined gray and white matter regions. All treated groups demonstrated successful viral transduction throughout the defined gray and white matter regions at −3.0 mm and +3.0 mm. control [high]-treated animals had, on average, higher levels of mRuby2 expression as compared to both NeuroD1-treated groups (Figure 1C.I–C.IV). In characterizing mRuby2+ cells, the mRuby2+/NeuN+ co-labeled cells were almost exclusively found in the gray matter regions (Figure 1C.I,C.III, magenta bars), with little co-labeling in the white matter regions (Figure 1C.II,C.IV, magenta bars). Interestingly, the number of mRuby2+/NeuN+ cells was consistent across all treatment groups. On the other hand, the control [high]-treated spinal cords generally had higher numbers of mRuby2+ cells co-labeled with GFAP, and the average number of mRuby2+/GFAP+ cells was consistent across gray and white matter regions at both −3.0 mm and +3.0 mm (Figure 1C.I–C.IV, green bars). Taken together, this demonstrates that the viral platform was successful in transducing spinal cells, and the transduced cells survived up to 6 WPC. However, while mRuby2+ cells expressed the NeuN neuronal marker in response to the NeuroD1 treatments, a number of mRuby2+ cells were found to express NeuN in response to the control [high] treatment as well. This suggests that the AAV9 DIO delivery system was not specific to targeting reactive astrocytes for reprogramming and that endogenous neurons were also seemingly transduced by the viral platform.

### 3.2. Titer-Dependent Deleterious Effect of Acute NeuroD1 Treatment on Spinal Cord Tissue

**Figure 2:**
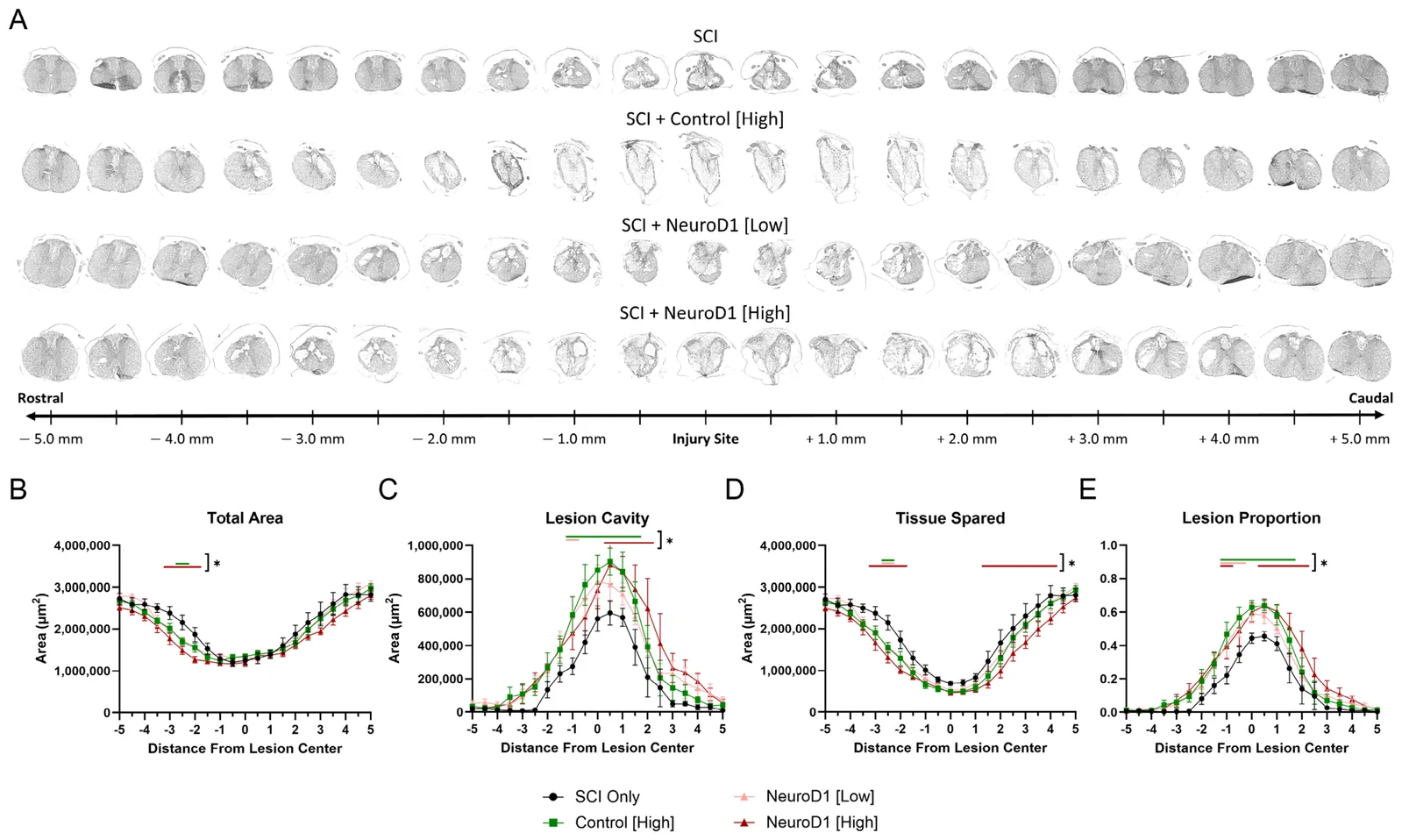
Spinal cord tissue analysis in response to acute NeuroD1 treatment. (**A**) Transverse sections of spinal cord tissue at 6 WPC from select spinal cords of each experimental treatment group extending 5.0 mm rostral and caudal to the lesion epicenter, at 500 µm intervals. Tissue was stained with hematoxylin and eosin. These sections were then analyzed for (**B**) total tissue area, (**C**) lesion cavity area, and (**D**) tissue spared, calculated by subtracting lesion cavity area from total tissue area. From these values, (**E**) the proportion of lesion was calculated by dividing the lesion cavity area by the total area. Measurements were grouped and assessed by treatment type, including SCI-only (black; *n* = 5), control [high] (green; *n* = 5), NeuroD1 [low] (light red; *n* = 5), and NeuroD1 [high] (dark red; *n* = 5). All data shown are presented as mean ± standard error. * *p* < 0.05 as comparisons from treatment group to the SCI-only group, two-way ANOVA followed by Tukey’s post hoc test.

Considering that NeuroD1 has been implicated in supporting axonal regeneration after sciatic nerve injury [39], we sought to evaluate the neuroprotective potential of the NeuroD1 treatment platform, regardless of the evidence suggesting both a lack of astrocyte-specific targeting and neuronal off-targeting by the viral delivery system. To do this, we assessed the effect of viral treatment on tissue preservation across the injury area. We stained the spinal cord tissue with H&E for anatomical visualization of the spinal cord structures. To measure the full spread of the lesion, we analyzed sections ranging from −5.0 mm to +5.0 mm, at 0.5 mm increments, relative to the injury center (Figure 2A). First, we measured the total area of each spinal cord section, and the measurements from control [high]- and both NeuroD1-treated groups were then evaluated in comparison to the measurements from SCI-only controls (Figure 2B). In response to NeuroD1 [high] treatment, there was a consistent decrease in total tissue area both rostral and caudal to the injury center, reaching significance from −2.0 mm to −3.0 mm (Figure 2B, dark red). Although not to the same extent as NeuroD1 [high] treatment, there was also a decrease in total tissue area rostral to the injury site, reaching significance at −2.5 mm, in response to control [high] treatment (Figure 2B, green). Despite generally lower average total tissue area, we found no significance between NeuroD1 [low] and SCI-only (Figure 2B, light red). We found similar trends when measuring the size and spread of the lesion cavity area (Figure 2C). NeuroD1 [high]- and control [high]-treated spinal cords had similarly increased lesion size and spread as compared to the untreated SCI-only group; the larger lesion size reached significance from 0 mm to +2.0 mm for NeuroD1 [high] treatment (Figure 2C, dark red) and −1.0 mm to +1.5 mm for control [high] treatment (Figure 2C, green). NeuroD1 [low] treatment also resulted in an increased lesion size, albeit at a lower peak than both high titer conditions, but this increase only reached significance at −1.0 mm (Figure 2C, light red). The final measurement, area of spared tissue, was calculated by subtracting the lesion cavity area from the total tissue area for each section (Figure 2D). NeuroD1 [high] treatment resulted in a significant decrease in spared tissue from −3.0 mm to −2.0 mm and from +1.5 mm to +4.0 mm (Figure 2D, dark red). Meanwhile, NeuroD1 [low]- and control [high]-treated spinal cords had consistently lower spared tissue area but only reached significance at −2.5 mm (Figure 2D, light red and green).

To account for any potential differences in spinal cord sizes between animals, we also quantified the proportion of total tissue area that was lesion cavity (Figure 2E). Here we found that all treated groups had a larger proportion of cavitated tissue as compared to untreated groups; significance for these treated groups were found at −1.0 mm and from +0.5 mm to +2.0 mm for NeuroD1 [high] (Figure 2E, dark red), from −1.0 mm to −0.5 mm for NeuroD1 [low] (Figure 2E, light red), and from −1.0 mm to +1.5 mm for control [high] (Figure 2E, green). Collectively, these results suggest that acute viral intervention had a deleterious effect on spinal cord tissue integrity. Furthermore, administration of the viruses at a high titer, particularly with NeuroD1 involvement, had significantly increased lesion cavity size and decreased tissue sparing.

### 3.3. Titer-Dependent Deleterious Effect of Acute Viral Treatment on Motor Functional Recovery

**Figure 3:**
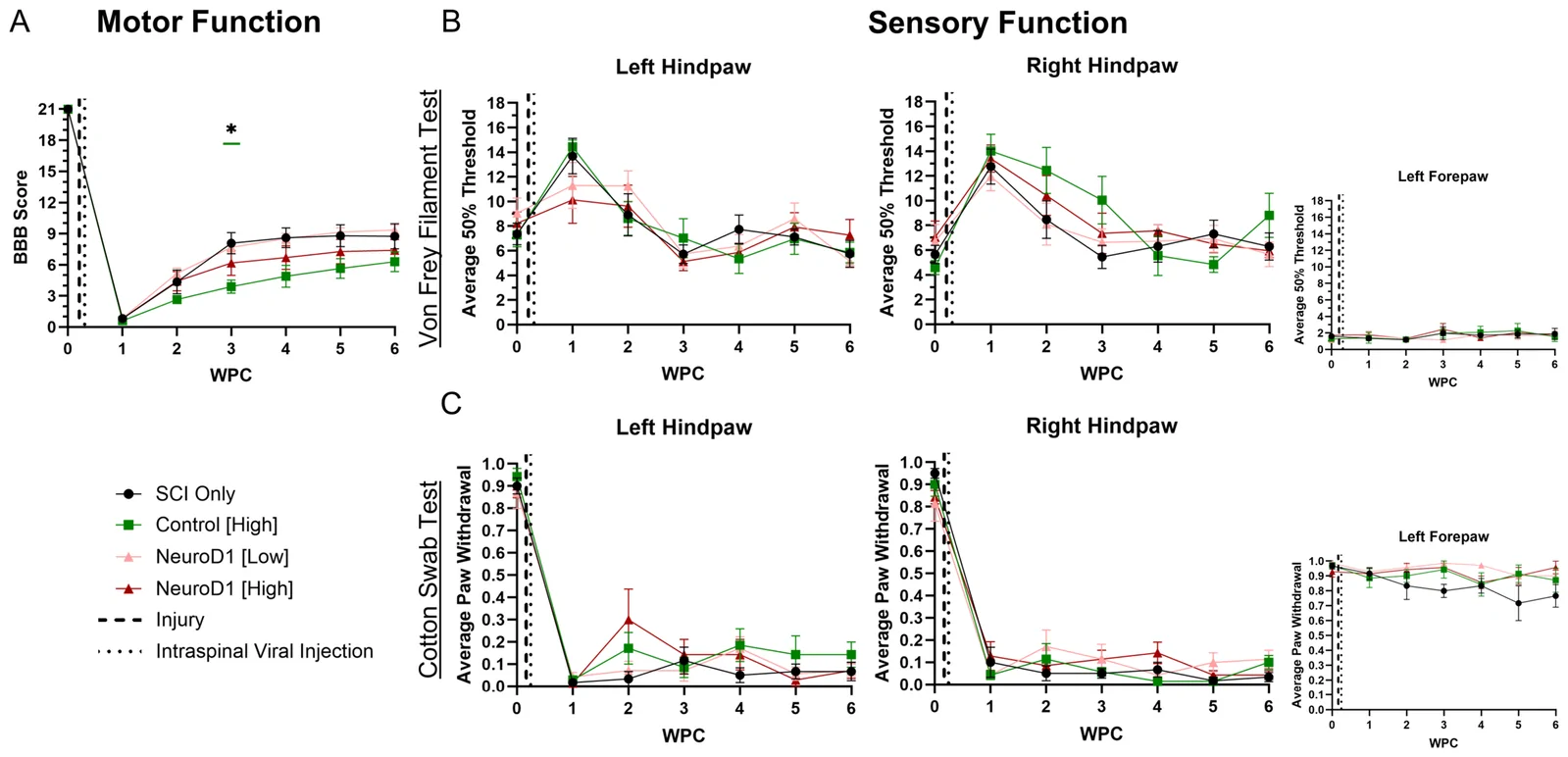
Effect of acute NeuroD1 treatment on functional recovery after moderate SCI. Following moderate SCI and subsequent viral treatment, motor and sensory functional recovery was assessed for 6 WPC. Motor function was analyzed using (**A**) BBB scoring, and sensory function was analyzed using (**B**) von Frey filament test and (**C**) cotton swab test. For sensory testing, the left forepaw was assessed as a non-injured control. Behavioral data was grouped and assessed by treatment type, including SCI-only (black circles; (**A**,**B**) *n* = 7, (**C**) *n* = 6), control [high] (green squares; (**A**–**C**) *n* = 7), NeuroD1 [low] (light red triangles; (**A**–**C**) *n* = 7), and NeuroD1 [high] (dark red triangles; (**A**,**B**) *n* = 9, (**C**) *n* = 7). Animals were excluded from analyses if their baselines were greater than 2 SD from the mean. All data shown are presented as mean ± standard error. * *p* < 0.05 as comparisons from treatment group to the SCI-only group, two-way ANOVA with repeated measures followed by Tukey’s post hoc test.

Considering the deleterious effect of viral treatment on tissue integrity, we aimed to determine whether this deleterious effect extended to motor and sensory function. Rats were evaluated in a series of weekly behavioral assays for 6 weeks after moderate contusion injury, in addition to pre-injury baselines (0 WPC). Motor function was assessed using BBB scoring (Figure 3A). Interestingly, we found that rats that received the control [high] treatment consistently scored lower than all other experimental groups throughout the assessment period, reaching significance at 3 WPC (Figure 3A, green). The NeuroD1 [high]-treated rats trended toward a worse motor performance than SCI-only, but did not reach significance (Figure 3A, light red). At 6 WPC, the average BBB scores for all conditions were 8.750 ± 1.215 for SCI-only (Figure 3A, black), 6.286 ± 0.950 for control [high] (Figure 3A, green), 9.357 ± 0.469 for NeuroD1 [low] (Figure 3A, light red), and 7.389 ± 1.041 for NeuroD1 [high] (Figure 3A, dark red). For SCI-only and NeuroD1 [low], their BBB scores translate to extensive movement in all three joints, plantar paw placement, and some instances of weight support. For NeuroD1 [high] treated rats, their average 6 WPC BBB score translates to extensive movement in all three joints, but no paw placement or weight support. For the control [high] treatment group, their average 6 WPC BBB score translates to extensive movement in two joints and slight movement in one joint.

To evaluate the effect of the acute viral treatment on sensory function after moderate SCI, we used the von Frey filament test (Figure 3B) and cotton swab test (Figure 3C). To account for any potential differences between hindpaws, we assessed each hindpaw separately. We also tested the left forepaw as a negative control, as the T8/T9 SCI does not affect forelimbs. Using the Up–Down method to measure the 50% paw withdrawal threshold for the von Frey test, there was no significant difference between the untreated SCI-only control and all viral treatment groups in either hindpaw (Figure 3B). Similarly, there were no significant differences in the average number of paw withdrawals from the cotton swab test between the SCI-only group and all viral treatment groups (Figure 3C).

### 3.4. mRuby2+ Cells Co-Label with Markers for Reactive Microglia/Macrophages

**Figure 4:**
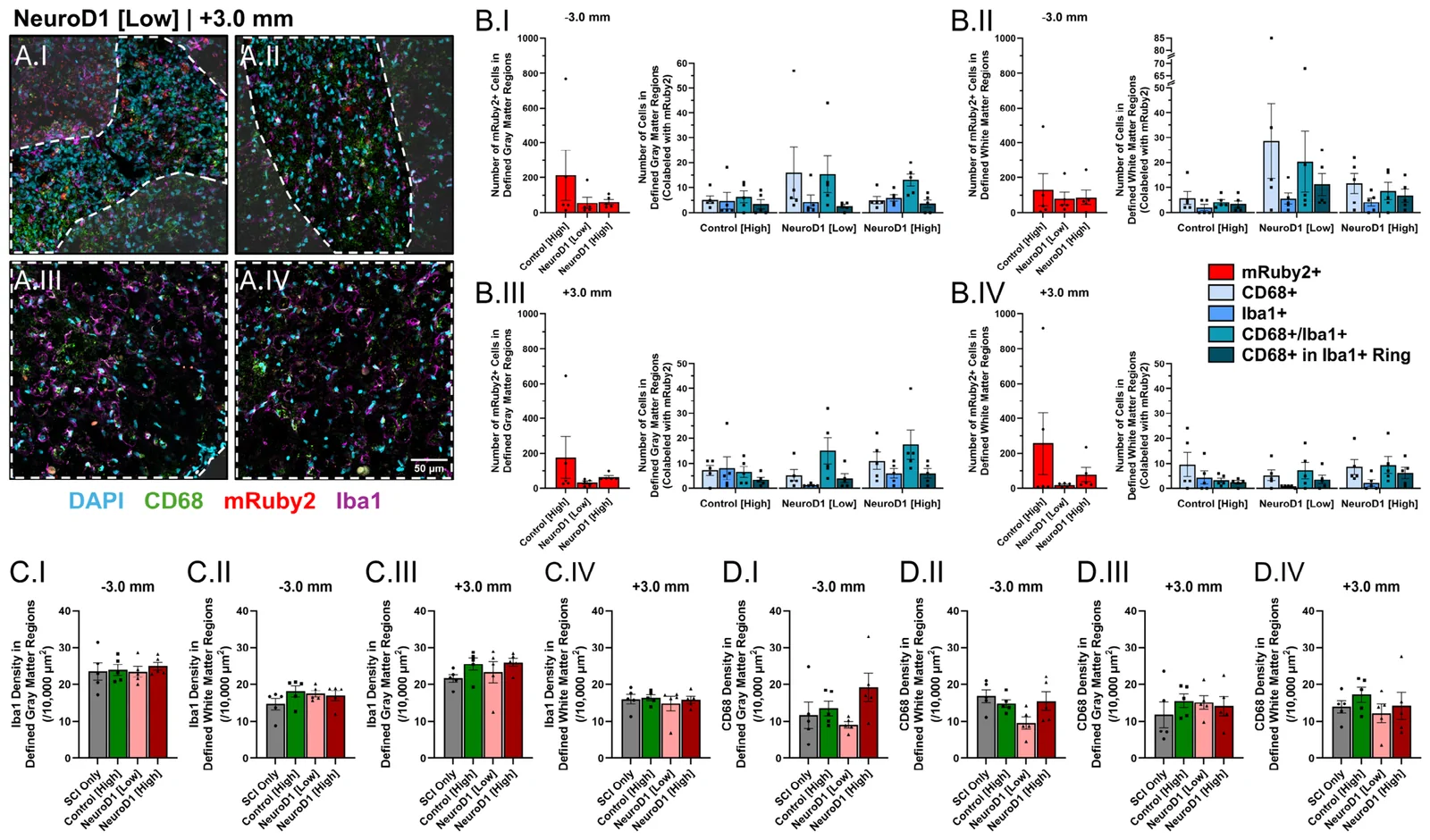
Effect of acute NeuroD1 treatment on neuroinflammation. IHC was performed on transverse sections of spinal cord at 3.0 mm rostral (−3.0 mm) and caudal (+3.0 mm) to the injury site, staining for CD68 (green), Iba1 (magenta), and counterstained with DAPI (cyan); mRuby2, the reporter protein for successful viral transduction, is shown in red. (**A.I**,**A.IV**) Representative 20× confocal images of a spinal cord section from a NeuroD1 [low]-treated spinal cord at +3.0 mm are shown. mRuby2 quantification and co-labeling analyses were then performed at 4 select locations: (**A.I**) central canal (**A.II**) ventral horn (**A.III**) ventromedial white matter, and (**A.IV**) ventrolateral white matter. (**B.I**–**B.IV**) Quantification of total mRuby2 expression and co-labeling with the neuroinflammatory markers at −3.0 mm (**B.I**,**B.II**) and +3.0 mm (**B.III**,**B.IV**) from the injury center (*n* = 5 for each experimental group). Defined gray matter and white matter regions are represented by combining the areas within the white dashed lines in (**A.I**,**A.II**) and (**A.III**,**A.IV**), respectively. (**C.I**–**C.IV**) Iba1 and (**D.I**–**D.IV**) CD68 densities in defined gray and white matter regions at −3.0 mm and +3.0 mm. All data shown are presented as mean ± standard error.

A significant consequence of SCI involves the cascading neuroinflammatory signaling throughout the stages of secondary injury. Therefore, we were interested in evaluating the effects of the viral NeuroD1 treatments on the spinal populations of reactive microglia and macrophages after injury. Since the GFAP and NeuN characterization of mRuby2+ cells revealed that astrocytes and neurons did not account for all mRuby2-expressing cells (Figure 1C.I–C.IV), we first wanted to evaluate co-labeling of mRuby2+ cells with markers for microglia and macrophages—Iba1 and CD68—in defined gray and white matter regions of the spinal cord at −3.0 mm and +3.0 mm (Figure 4A.I–A.IV,B.I–B.IV). Here we found four patterns of Iba1 and CD68 co-labeling with mRuby2: Iba1 alone, CD68 alone, both CD68 and Iba1, and CD68 within an Iba1 ring (Figure 4B.I–B.IV). Broadly, we found evidence of co-labeling between mRuby2 and the microglia/macrophage markers in response to control [high], NeuroD1 [low], and NeuroD1 [high] treatments at all assessed locations. Interestingly, co-labeling of mRuby2 with CD68 and Iba1 account for substantial portion of mRuby2-expressing cells in both the NeuroD1 [low]- and NeuroD1 [high]-treated groups within the defined gray and white matter regions as compared to control [high]-treated group (Figure 4B.I–B.IV).

### 3.5. NeuroD1 Treatment Has No Effect on Reactive Microglia/Macrophage Density

NeuroD1, when used in the context of astrocyte-to-neuron reprogramming, has also been associated with a reduction in neuroinflammation, as measured by markers of reactive microglia and macrophages [24]. Therefore, we were interested in evaluating the density of Iba1- and CD68-expressing cells to determine whether NeuroD1 treatments had any effect on overall levels of neuroinflammation. The density of Iba1+ cells (Figure 4C.I–C.IV) and CD68+ cells (Figure 4D.I–D.IV) in defined gray matter and white matter regions was quantified at −3.0 mm and +3.0 mm. Here we found no significant differences between viral treatment groups and the untreated SCI-only group in Iba1 density (Figure 4C.I–C.IV) and CD68 density (Figure 4D.I–D.IV), regardless of location measured.

### 3.6. Correlations with BBB Scores

With all of the analyses we conducted on mRuby2 characterization, tissue dynamics, and neuroinflammation in response to the viral treatments, we sought to establish the relationship between these measurements and the BBB scores. To achieve this, we performed a Pearson correlation between the BBB scores at 6 WPC and the following factors at select distances from lesion center: total lesion area, lesion cavity area, spared tissue area, mRuby2 expression, Iba1 density, and CD68 density. The Pearson correlation coefficient and corresponding *p*-value are recorded in Table 1 and graphically represented, with linear regression, in Figure S2. We found a strong significant inverse correlation between the BBB scores and lesion cavity area (Table 1, Figure S2C) as well as spared tissue area (Table 1, Figure S2D) at all assessed distances: −3.0 mm, −1.5 mm, 0 mm, +1.5 mm, and +3.0 mm. There was a significant correlation between BBB scores and the total tissue area measurements −3.0 mm, −1.5 mm, and +3.0 mm (Table 1, Figure S2B). Interestingly, there was no correlation between BBB scores and mRuby2 expression (Table 1, Figure S2A) or Iba1 density (Table 1, Figure S2E). For CD68 density, there was a significant correlation to BBB scores at −3.0 mm only (Table 1, Figure S2F).

### 3.7. AAV9-NeuroD1 Platform Successfully Transduces Spinal Cells When Administered in the Subacute Stage of Injury

**Figure 5:**
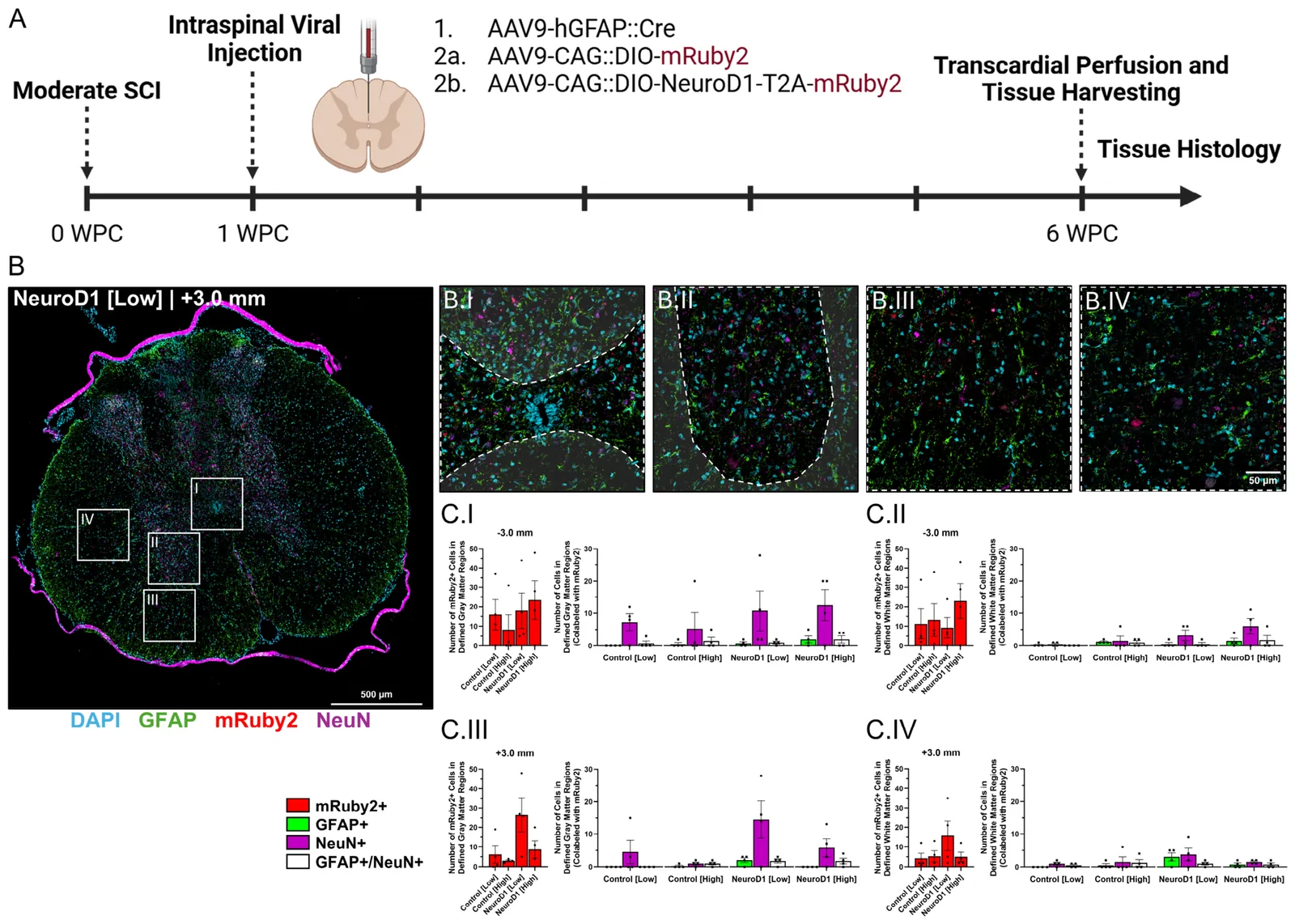
NeuroD1 transduction in the spinal cord after subacute intervention. (**A**) Rats were given a moderate contusion SCI at the T8/T9 level. At 1 WPC, the rats received an intraspinal injection of the AAV9 treatment platform at one of two titers, resulting in four treatment groups. control-treated rats received hGFAP::Cre and CAG::DIO-mRuby2 at a low and high titer—control [low] and control [high], respectively. NeuroD1-treated rats received hGFAP::Cre and CAG::DIO-NeuroD1-T2A-mRuby2 at a low and high titer—NeuroD1 [low] and NeuroD1 [high], respectively. An additional control group, SCI-only, remained untreated. IHC was used to stain spinal cord tissue with GFAP (green), NeuN (magenta), and counterstained with DAPI (cyan). The viral reporter protein, mRuby2, is shown in red. A representative spinal cord section from a NeuroD1 [low]-treated spinal cord at 3.0 mm caudal to the injury site is shown. mRuby2 expression and co-labeling quantification were assessed at two gray matter regions, (**B.I**) central canal and (**B.II**) ventral horn, and two white matter regions, (**B.III**) ventromedial and (**B.IV**) ventrolateral white matter, of spinal cord sections at −3.0 mm and +3.0 mm. Quantification of mRuby2 expression and characterization from the defined gray matter (**C.I**,**C.III**) and white matter regions (**C.II**,**C.IV**), as measured by combining the areas within the dashed lines in (**B.I**,**B.II**) and (**B.III**,**B.IV**), respectively, at −3.0 mm and +3.0 mm, All data shown are presented as mean ± standard error.

Given the deleterious motor and tissue effects observed with acute intervention, we next evaluated the AAV9-NeuroD1 platform when administered in the subacute stage of injury. The viral treatments—control [low], control [high], NeuroD1 [low], and NeuroD1 [high]—were administered directly into the spinal cord at 1 WPC and spinal cords were extracted for histological analysis at 6 WPC (Figure 5A,B). Interestingly, when assessing the efficacy and specificity of viral transduction under the same parameters as with our acute treatment histological analysis, we found evidence of successful transduction by way of mRuby2 expression, albeit at relatively lower levels than observed with acute treatment (Figure 5C.I–C.IV, red bars). All treatment groups displayed a wide range of mRuby2 expression, with some selected tissue sections not showing any mRuby2 expression in the selected gray and white matter regions of interest. Generally, NeuroD1-treated groups displayed a higher number of mRuby2-expressing cells than control-treated groups. We then evaluated the identity of the mRuby2+ cells by co-labeling with NeuN and GFAP, where we found that a majority of mRuby2+ cells were co-labeled with NeuN in the gray matter regions (Figure 5C.I,C.III). While there was some evidence of mRuby2+/NeuN+ cells in the white matter regions of the spinal cord, NeuN and GFAP do not fully capture the breadth of mRuby2-expressing cells in the white matter (Figure 5C.II,C.IV). Interestingly, mRuby2/GFAP co-labeling was limited across all analyzed regions (Figure 5C.I–C.IV, green bars). We also observed mRuby2+/NeuN+ cells in both NeuroD1- and control-treated spinal cord sections, independent of viral titer (Figure 5C.I–C.IV, magenta bars). Nonetheless, NeuroD1-treated groups displayed higher numbers of mRuby2+/NeuN+ cells than control-treated groups. Taken together, these results show that the AAV9-NeuroD1 reprogramming platform was capable of transducing spinal cells that survived up to 5 weeks after viral intervention, but direct evidence of astrocyte-to-neuron reprogramming could not be determined from histological analysis alone due to observed neuronal off-targeting.

### 3.8. High Titer Subacute NeuroD1 Treatment Preserves Spinal Cord Tissue

**Figure 6:**
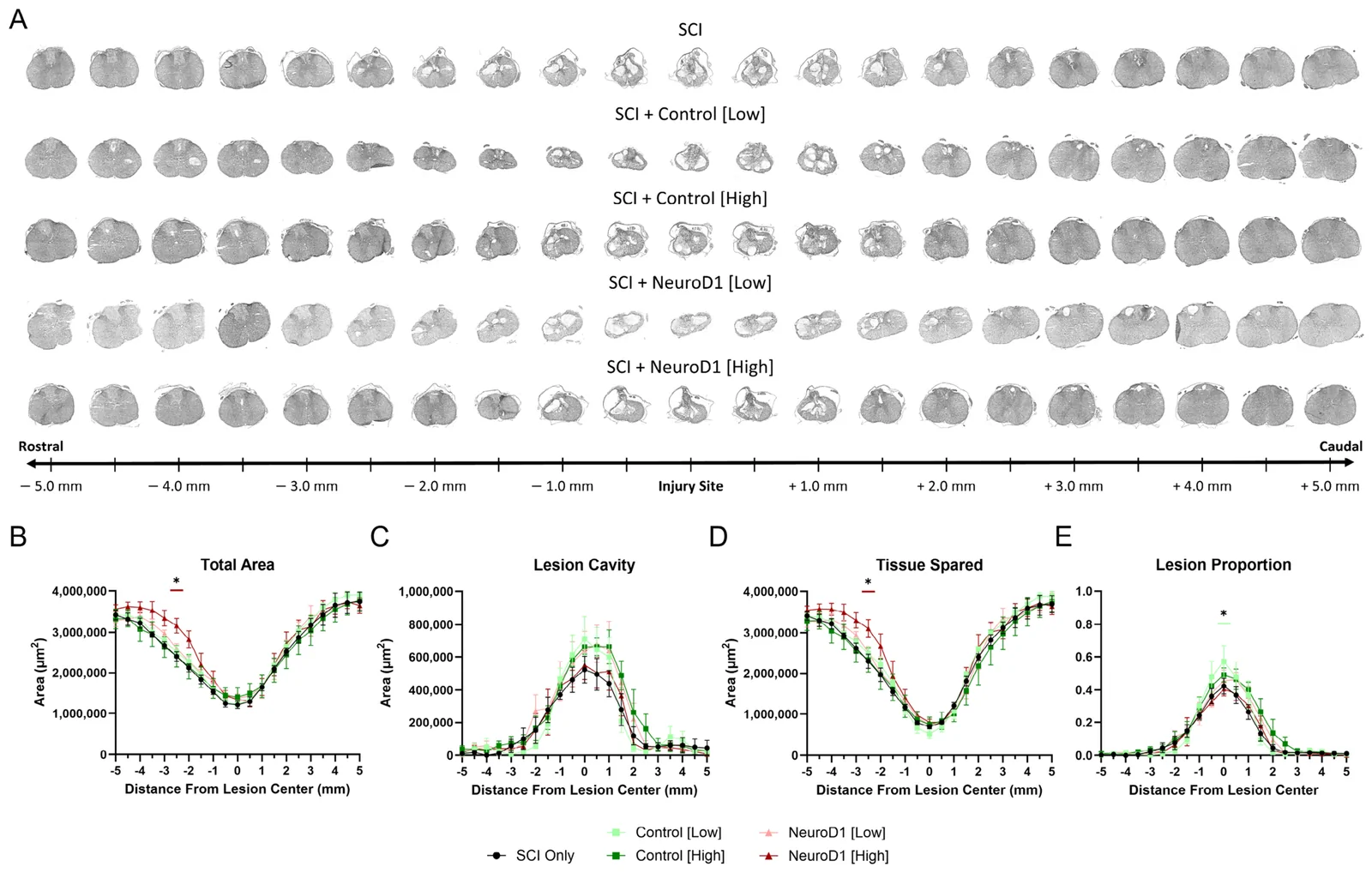
Effect of subacute NeuroD1 treatment on spinal cord tissue. (**A**) H&E stains of transverse spinal cord sections at 6 WPC from each experimental treatment group: SCI-only, control [low], control [high], NeuroD1 [low], and NeuroD1 [high]. The tissue was assessed at 500 µm increments, extending from −5.0 mm to +5.0 mm. The area of (**B**) total tissue and (**C**) lesion cavity was measured for each spinal cord section. (**D**) The area of tissue spared was calculated by subtracting the lesion cavity area from the total tissue area of each section. (**E**) The lesion proportion was calculated by dividing the lesion cavity area from the total area. (**B**–**E**) Average values for each treatment group are shown: SCI-only (black; *n* = 4), control [low] (light green; *n* = 4), control [high] (dark green; *n* = 4), NeuroD1 [low] (light red; *n* = 4), and NeuroD1 [high] (dark red; *n* = 4). All data shown are presented as mean ± standard error. * *p* < 0.05 as comparisons from treatment group to the SCI-only group, two-way ANOVA followed by Tukey’s post hoc test.

Considering that we have previously demonstrated a titer-dependent adverse effect of the viral treatment on the spread of the lesion cavity and the extent of spared tissue when administered in the acute stage of injury (Figure 2), we were interested in evaluating the titer-dependent effects of subacute NeuroD1 treatment on tissue integrity. Tissue sections spanning −5.0 mm to +5.0 mm, at 0.5 mm intervals were stained with H&E and subsequently analyzed for tissue and lesion cavity areas (Figure 6A). We found that NeuroD1 [high] treatment resulted in an increase in the average total tissue area rostral to the injury site, reaching significance at −2.5 mm, compared to the SCI-only group (Figure 6B, dark red). There were no significant differences in total tissue area in response to all other treatments. When we measured the area of the lesion cavity in each section across the injury site, we found a non-significant increase in lesion cavity size near the injury center in response to control [low] (Figure 6C, light green), control [high] (Figure 6C, dark green), and NeuroD1 [low] (Figure 6C, light red) treatments; NeuroD1 [high] did not appear to have any effect on lesion cavity size (Figure 6C, dark red). After calculating the area of spared tissue in each spinal cord section, NeuroD1 [high]-treated spinal cords exhibited increased amounts of spared tissue rostral to the injury center, reaching significance at −2.5 mm (Figure 6D, dark red). There was no significant effect on spared tissue area in response to the other three treatments. Finally, we calculated the proportion of cavitated tissue where both control [low] and control [high] treatments appeared to increase the proportion of lesioned tissue around the injury epicenter, but only control [low] reached significance at 0 mm (Figure 6E). Meanwhile, NeuroD1 [high] treatment did not appear to have any effect on lesion proportion (Figure 6E, dark red).

Although we did not find any effect of acute viral treatment on activated microglia/macrophage population density, we were interested in determining whether this persisted for subacute intervention. We measured Iba1+ and CD68+ cell density in white and gray matter regions of tissue sections at −3.0 mm and +3.0 mm. There was no significant effect of viral treatment, control or NeuroD1, on Iba1 density (Figure S3A.I–A.IV) or CD68 density (Figure S3B.I–B.IV).

### 3.9. Subacute AAV9-NeuroD1 Treatment Has No Effect on Motor or Sensory Functional Recovery

**Figure 7:**
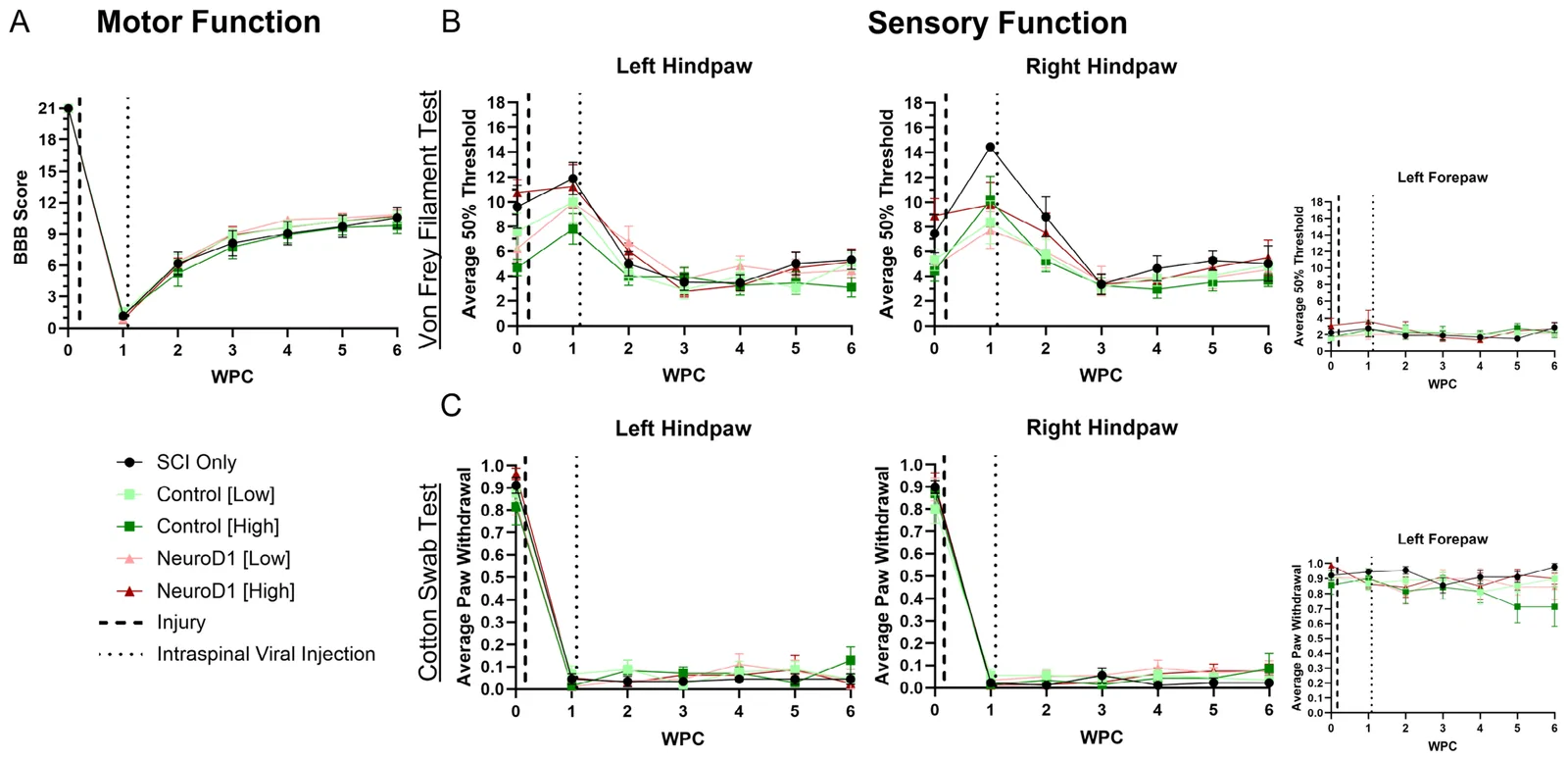
Effect of subacute NeuroD1 treatment on motor and sensory functional recovery. Motor and sensory function were evaluated weekly up to 6 WPC, including a pre-injury baseline (0 WPC). At 1 WPC, motor and sensory evaluation occurred prior to viral intervention. (**A**) BBB scoring was used to assess motor function. (**B**) The von Frey filament test and (**C**) cotton swab test were used to assess sensory function. Behavioral data was grouped and assessed by treatment. The averages within each treatment group are shown: SCI-only (black circles; (**A**–**C**) *n* = 9), control [low] (light green squares; (**A**) *n* = 11, (**B**,**C**) *n* = 9), control [high] (dark green squares; (**A**) *n* = 8, (**B**,**C**) *n* = 7), NeuroD1 [low] (light red triangles; (**A**,**B**) *n* = 11, (**C**) *n* = 9), and NeuroD1 [high] (dark red triangles; (**A**,**B**) *n* = 10, (**C**) *n* = 8). Animals were excluded from analyses if their baselines were greater than 2 SD from the mean. All data shown are presented as mean ± standard error. Two-way ANOVA with repeated measures followed by Tukey’s post hoc test was performed.

Ultimately, the AAV9-NeuroD1 treatment platform is intended to restore nervous system function after SCI. Given that acute viral intervention resulted in a worse motor performance, we were interested in investigating the motor and sensory functional recovery after subacute intervention. Surprisingly, BBB scoring revealed no significant effect of viral treatment on motor function, regardless of titer (Figure 7A). At 6 WPC, the average BBB score for each treatment group was 10.556 ± 1.005 for SCI-only (Figure 7A, black), 10.591 ± 0.595 for control [low] (Figure 7A, light green), 9.813 ± 0.750 for control [high] (Figure 7A, dark green), 10.864 ± 0.443 for NeuroD1 [low] (Figure 7A, light red), and 10.700 ± 0.611 for NeuroD1 [high] (Figure 7A, dark red). These BBB scores translate to extensive movement in all three joints, plantar paw placement, and occasional weight support with no hindlimb coordination. These results demonstrate that the viral treatment had no effect on motor functional recovery post-SCI, regardless of titer. Similarly, when we evaluated sensory function in response to the viral treatments, the von Frey filament test and cotton swab test revealed no significant differences in 50% paw withdrawal thresholds (Figure 7B) and paw withdrawal responses (Figure 7C), respectively.

## 4. Discussion

In vivo astrocyte-to-neuron reprogramming is an emerging experimental therapeutic approach that makes use of neurogenic transcriptions factors to selectively transdifferentiate astrocytes into neurons [16]. The astrocyte-to-neuron reprogramming platform has been studied with the use of several different neurogenic transcription factors for a variety of CNS injuries and disorders [17,36,37,40,41,42,43,44,45]. While these studies have shown evidence of successful reprogramming, others have questioned the viability of the in vivo reprogramming platform by providing results demonstrating a lack of specificity in astrocyte targeting due to neuronal off-targeting [28]. Nevertheless, the extent to which the AAV9-NeuroD1 platform can promote functional recovery and neuroprotective benefit, particularly for SCI, remains a valuable area of research. In this present study, we evaluated the dose-dependent and intervention-dependent therapeutic potential of the AAV9-NeuroD1 DIO reprogramming platform for moderate T8/T9 SCI in rats.

### 4.1. The AAV9-NeuroD1 DIO Delivery Platform Successfully, but Non-Specifically, Transduces Spinal Cells

We show here that the AAV9-based DIO viral delivery platform was capable of transducing cells in the gray and white matter of the spinal cord when administered in the acute stage of injury (Figure 1) and the subacute stage of injury (Figure 5) at both the low and high titers of 10^11^ GC/mL and 10^13^ GC/mL, respectively. In response to acute administration, control [high] treatment was demonstrated to have a higher average number of mRuby2+ cells as compared to NeuroD1 [low] and NeuroD1 [high] treatments. However, our exploratory histological analysis also revealed substantial variation in the levels of viral transduction within treatment groups; sections from two rats in the control [high] treatment group displayed extensive mRuby2 expression as compared to the lower number of mRuby2+ cells in the sections from the remaining three rats—which were more consistent with the lower levels of mRuby2 expression from both NeuroD1 treatment groups. It is also important to note that our study design did not include an acute control [low] treatment condition, thus limiting our titer-dependent evaluation of mRuby2 expression and variability. Regardless, we observed similar variability in viral transduction for subacute intervention, albeit to a lesser extent primarily due to the unexpected low overall transduction level across all subacute treatment conditions. While these differences in mRuby2 expression levels between treatment and intervention groups may be explained by a low transduction efficiency of the viral delivery system, it is also possible that the structure of the viral construct itself affected mRuby2 expression; research has shown that the use of 2A peptides can result in reduced protein expression at the second position—mRuby2, for this study—in the viral genome [46]. Therefore, it is possible that the reduced mRuby2 expression in NeuroD1-treated spinal cords does not correlate to a reduction in successful NeuroD1 transduction. Although we found mRuby2/NeuN co-labeling in response to acute and subacute NeuroD1 treatments, which would normally imply successful astrocyte-to-neuron reprogramming, there was also evidence of mRuby2/NeuN co-labeling in control-treated spinal cords. This indicates that endogenous neurons were seemingly also transduced by the reprogramming platform, ultimately suggesting a lack of astrocyte-specific targeting. This is consistent with previous studies that have demonstrated non-specific in vivo viral transduction using this platform [28]. As a result of this neuronal off-targeting, the results discussed here cannot be attributed to ‘reprogramming.’ The prevailing hypotheses to explain this neuronal leakage involve the non-specificity of the AAV DIO delivery platform and the use of a high viral titer.

AAV is a virus that infects both dividing and non-dividing cells [47,48]. While wild-type AAV can integrate into the human genome, recombinant AAV lack the genes necessary for genome integration, thus conferring a reduced integration risk [48,49]. As a result, AAV vectors that are commonly used in preclinical and clinical studies are capable of persistent episomal expression in non-dividing cells and gradually diminishing expression in dividing cells [47,48]. This episomal persistence and lack of host genome integration makes AAV delivery systems suitable for long-term gene therapy approaches, particularly those that target non-dividing cells, such as neurons. However, this ability to target both dividing and non-dividing cells may not be ideal for astrocyte-specific targeting in the rat spinal cord, regardless of its clinical advantages. This concern is furthered by AAV serotype tropism and tropism-associated “leakiness”; each AAV serotype confers a particular tropism for a type of cell or tissue it preferentially infects [47,50]. AAV9 in particular has a broad tropism, able to infect a wide range of CNS cells in addition to peripheral tissue [50,51]. Therefore, it is possible that the use of the AAV9 serotype in this study may have contributed to the observed neuronal leakage. Future studies may be interested in other serotypes, such as AAV5 and AAV8, that have a tropism conducive to astrocyte targeting while potentially limiting neuronal leakage. Alternatively, a recent study has highlighted AAV11, in combination with the GfaABC_1_D promoter, as an enhanced method of astrocyte viral transduction [52]. Alternatively, it may be advantageous to use retroviruses or lentiviruses in place of AAVs. Lentiviruses have a lower risk of neuronal leakage due to select envelope protein modification for targeted transduction [53]. On the other hand, retroviruses only transduce dividing cells [54], which would eliminate all concern for neuronal off-targeting. While lentiviruses and retroviruses confer separate risk, namely insertional mutagenesis [53,54], these viruses may be considered to mitigate unintentional neuronal off-targeting in studies aimed at evaluating the therapeutic efficacy of single-factor, in vivo astrocyte-to-neuron reprogramming approaches.

Several studies have reported on titer-dependent leakiness associated with AAV-based, Cre-dependent delivery approaches [24,29,30]. While there is a minor titer-independent risk of Cre-independent spontaneous recombination [55], using a titer on the magnitude of 10^13^ GC/mL confers additional risk of neuronal leakage [24,30]. One possible explanation for this high titer leakage is the formation of complex concatemer structures within the nucleus [55]. With a high viral titer, there may be an increased likelihood of multiple AAV genomes existing in the episomal state within a cell, thus allowing for concatemer formation; considering the different configurations of concatemer formation, it is possible for a configuration in which the CAG promoter from one genome allows for the translation of the inverted NeuroD1 sequence from another genome. Another possible explanation concerns the broad tropism of AAV9 [50,51], where increasing the viral titer would increase the likelihood of off-targeting due to overwhelming viral presence. The titer-dependent leakiness has influenced astrocyte-to-neuron reprogramming researchers to adopt lower viral titers—on the magnitude of 10^11^–10^12^ GC/mL [24,30,38]—while making use of other tools, such as a CMV enhancer [38], to drive gene expression. While these proposed changes to AAV-based reprogramming delivery platforms have not explicitly been outlined for SCI, future studies should also seek to use the viruses at a titer of 10^11^–10^12^ GC/mL while continuing to evaluate titer as a significant factor contributing to reprogramming efficiency. Ultimately, considering the substantial variability and non-specificity in viral transduction with the AAV9-based DIO delivery platform, subsequent studies should critically evaluate and optimize alternative delivery approaches to enhance target specificity, minimize viral load, and drive consistent gene expression to properly evaluate the therapeutic potential and viability of AAV9-NeuroD1-mediated astrocyte-to-neuron reprogramming.

Regardless, our results underscore the inherent ambiguity associated with relying solely on histological identification of fluorescent viral reporter proteins for confirming successful astrocyte-to-neuron conversion. While we were able to identify cells that were transduced by the viral platform by mRuby2 expression at 6 weeks post-intervention, this was insufficient in determining the cellular origin of mRuby2-labeled cells since both NeuroD1 and control treatments produced mRuby2-expressing, NeuN+ cells. Future AAV9-NeuroD1 reprogramming studies intending to assess the validity of the reprogramming platform should consider designing experiments that provide sufficient evidence establishing the cellular origin of the ‘reprogrammed cell.’ The prevailing approach for validating the astrocyte-to-neuron conversion is lineage tracing to label astrocytes prior to viral transduction [56,57]. Whether through the use of thymidine analogs [19,28] or transgenic animal models [28], histological identification of viral reporter-labeled cells supplemented by lineage tracing is crucial for demonstrating successful conversion of astrocytes into neurons in vivo.

### 4.2. The NeuroD1 Treatment Platform Offers Titer- and Intervention Timing-Dependent Deleterious Effects on Functional Recovery After SCI

Several studies that have used NeuroD1 to reprogram astrocytes into neurons have reported varying degrees of functional benefit. Researchers have demonstrated motor [36,40,41,42,43], visual [37], and neurocognitive improvement in animal stroke models as well as motor functional improvement in a Huntington’s Disease mouse model [44]. With respect to SCI, one study found motor functional improvement after acute lentiviral administration of NeuroD1 [58]. Surprisingly, our study found that acute administration of AAV9-NeuroD1 had a titer-dependent deleterious effect on lesion cavity size and spread (Figure 2C), tissue sparing (Figure 2D), and motor function (Figure 3A). This is of particular interest because there have not been any reports of AAV9-NeuroD1-mediated adverse effects on motor function or tissue dynamics, especially within the context of SCI. It should be noted that the demonstrated deleterious effects on motor function appear to be largely driven by the high titer of the viral delivery platform, as both control [high] and NeuroD1 [high] viral treatments were both associated with lower BBB scores. Acute treatment also demonstrated titer-dependent degrees of deleterious effect on lesion cavity area and tissue sparing. Interestingly, these results did not translate to subacute intervention where viral treatment had no effect on lesion cavity size (Figure 6C) or motor function (Figure 7A), but NeuroD1 treatment did manage to show a titer-dependent beneficial effect on tissue sparing after injury (Figure 6D). However, we did not observe any titer- or intervention timing-dependent effect on sensory function (Figure 3B,C and Figure 7B,C) or neuroinflammation (Figure 4C.I–C.IV,D.I–D.IV and Figure S3A.I–A.IV,B.I–B.IV). These findings are surprising for the field of NeuroD1-mediated astrocyte-to-neuron reprogramming, as previous studies have either demonstrated moderate to no functional benefit or did not have motor function or lesion and tissue area as outcome measures. Accordingly, the results demonstrated here offer new insight into the potential dose-dependent negative consequences of this viral reprogramming platform for SCI, particularly for acute intervention.

When we assessed the relationship between the lesion cavity area and BBB scores in response to acute intervention, we found that larger lesion cavity area was significantly correlated with lower BBB scores (Table 1, Figure S2C). Conversely, we found that increased tissue sparing was significantly correlated with higher BBB scores (Table 1, Figure S2D). While these correlations do not explain the cause of the treatment-related motor deficits, they do offer insight into the potential detrimental effects of acute viral treatment. Since the two vector AAV9 DIO delivery system is meant to target reactive astrocytes immediately after injury, a major concern with this platform is the potential to disrupt essential processes of resident and reactive astrocytes in the early stages of injury, namely reactive gliosis, astrocyte-mediated neuroinflammation, and glial scar production [5,13,27]. Seeing as the glial scar is responsible for sequestering toxic debris, limiting immune cell infiltration, and containing the cystic cavity [13,27], disrupting reactive astrocyte function during early stages of SCI pathology may contribute to the observed acute intervention-specific increased lesion spread and decreased tissue sparing. Additional studies should prioritize understanding the effect of acute AAV9-NeuroD1 treatment on resident reactive astrocyte function and glial scar formation throughout the early stages of injury along with uncovering the mechanisms underlying the viral treatment-mediated motor deficits.

### 4.3. Viability of NeuroD1 Reprogramming for SCI

While our study did provide evidence of successful transduction of spinal cells that remained present up to 6 WPC, we also observed intervention timing-independent co-labeling between mRuby2 and reactive microglia/macrophage markers Iba1 and CD68 (Figure 4 and Figure S3). It should be noted that Iba1 and CD68 can be expressed by both reactive microglia and macrophages, particularly after injury-related activation [59], so all combinations of Iba1 and CD68 co-labeling with mRuby2 are associated with reactive microglia and macrophages broadly in this exploratory histological analysis. Considering that reactive microglia and macrophages engage in phagocytic activity after injury [5], these results indicate that a substantial portion of the mRuby2+ cells have been engulfed by phagocytic reactive microglia/macrophages. This is consistent with two astrocyte-to-neuron reprogramming studies, one of which used NeuroD1 for cervical SCI and observed expression of their GFP reporter within cells of macrophage morphology [45,60]. This may be explained by reactive microglia and macrophages clearing virally transduced cells in a stage of injury that is characterized by neuroinflammation, reactive gliosis, and phagocytic activity [5]. However, it is important to recognize that phagocytic microglia and macrophages are involved in synaptic pruning and removal of dysfunctional or non-integrated neurons after injury [61,62] and during developmental neurogenesis [63,64]. Therefore, it is also possible that some astrocytes were indeed reprogrammed into neurons but were either immature or did not form functional synapses and were thus cleared by reactive microglia and macrophages. Future research should continue to assess the viability and enhance long-term survival of NeuroD1-transduced cells.

Finally, to fully assess the viability of AAV9-NeuroD1-mediated astrocyte-to-neuron reprogramming in vivo, researchers should consider potential neurotoxic effects resulting from high titer AAV administration. High AAV doses have been linked to cytotoxicity, blood–brain barrier disruption, leukocyte infiltration, and neurodegeneration in vivo [65,66,67,68]. As such, investigating markers of AAV-related neurotoxicity—such as apoptosis, T lymphocyte infiltration, and the development of anti-AAV antibodies—is essential for evaluating and establishing safety. Although assessing AAV neurotoxicity was outside the scope of this study, future studies interested in assessing the overall viability of this platform should consider safety evaluation as a component of study design to generate a comprehensive understanding of this treatment platform.

## 5. Conclusions

While our study demonstrates successful transduction of spinal cells that persisted up to 6 WPC, we also observed evidence of neuronal off-targeting. As a result, interpretations of these results reflect the effects of the viral treatment more broadly rather than the intended astrocyte-to-neuron reprogramming. We further report unexpected titer- and intervention timing-dependent deleterious effects on motor recovery, lesion cavity size and spread, and the extent of spared tissue. Ultimately, this AAV9-NeuroD1 DIO-based reprogramming treatment platform does not appear viable as an acute treatment for moderate SCI. However, subacute intervention showed promise in preserving spinal cord tissue, warranting continued study. Although prior NeuroD1-mediated studies have provided promising results that have led to optimistic outlooks as an experimental therapeutic platform, our findings underscore the need for caution regarding the viability of the viral platform, particularly for acute intervention. Additional research into the potential cytotoxic effects of the AAV9 delivery vehicle and optimization of the gene delivery strategy for subacute intervention is necessary to enhance reprogramming efficiency, limit neuronal off-targeting, and promoting functional and neuroprotective benefits for SCI recovery.

## Figures and Tables

**Table 1:** Correlations with BBB scores at defined distances from injury site.

Factor		−3.0 mm	−1.5 mm	0 mm	+1.5 mm	+3.0 mm
mRuby2 expression ^#^	Pearson Correlation	−0.06335				−0.04449
	*p*-value	0.8225				0.8749
Total tissue area	Pearson Correlation	0.5678	0.5627	−0.1672	0.2058	0.4733
	*p*-value	0.0078 *	0.0098 *	0.4811	0.3841	0.0350 *
Lesion cavity area	Pearson Correlation	−0.6344	−0.5792	−0.5119	−0.8099	−0.5571
	*p*-value	0.0027 *	0.0074 *	0.0211 *	<0.0001 *	0.0107 *
Spared tissue area	Pearson Correlation	0.6399	0.7242	0.6138	0.7159	0.5837
	*p*-value	0.0024 *	0.0003 *	0.0040 *	0.0004 *	0.0107 *
Iba1 density	Pearson Correlation	0.01441				−0.3483
	*p*-value	0.9519				0.1213
CD68 density	Pearson Correlation	−0.4903				−0.3189
	*p*-value	0.0282 *				0.1705

^#^ Total number of mRuby2+ cells across defined gray and white matter regions, as measured in Figure 1. * Correlation is significant at the 0.05 level (2-tailed).

## Data Availability

The original contributions presented in this study are included in the article/Supplementary Materials. Further inquiries can be directed to the corresponding authors.

## Supplementary Materials

The following supporting information can be downloaded at: https://www.mdpi.com/article/10.3390/cimb48080786/s1.

## Abbreviations

The following abbreviations are used in this manuscript:

SCI	Spinal Cord Injury
CNS	Central Nervous System
AAV	Adeno-Associated Virus
WPC	Weeks Post-Contusion
GC/mL	Genome Copies per mL
BBB	Basso, Beattie, and Bresnahan
H&E	Hematoxylin and Eosin
IHC	Immunohistochemistry
EDF	Extended Depth of Focus
ANOVA	Analysis of Variance
