# Supplementary material for: Titer- and Intervention Timing-Dependent Functional Effects of AAV9-NeuroD1 Gene Therapy on Spinal Cord Injury
Source: Curr Issues Mol Biol. 2026 Aug 2;48(8):786. doi: 10.3390/cimb48080786 (PMC13511481; doi:10.3390/cimb48080786)
Supplement: Supplementary file 1 [file cimb-48-00786-s001.zip › cimb-4405455-supplementary.pdf]

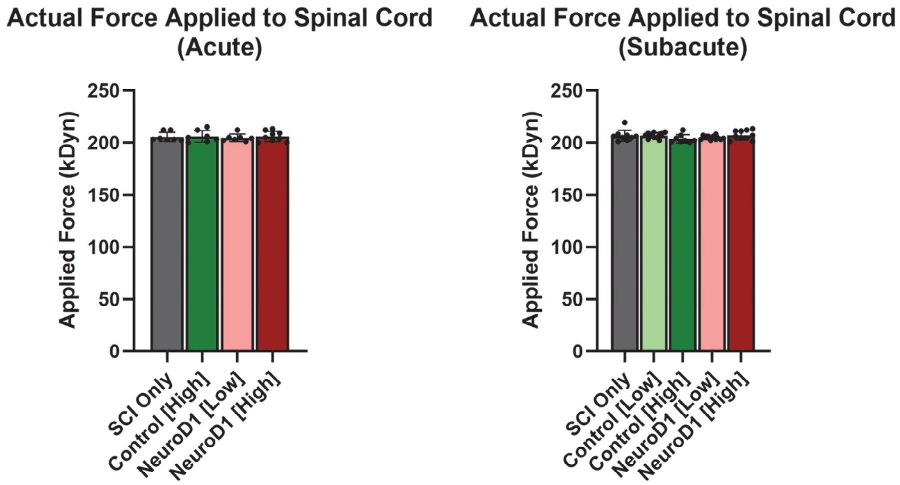

Figure S1. Force Applied to Spinal Cords for Moderate Contusion Spinal Cord Injury. Recorded force applied to spinal cords to create a moderate contusion SCI at the thoracic 8/9 level of the spinal cord. The average force applied to spinal cords for acute treatment were 205.571 ± 4.429 for SCI Only, 206.000 ± 5.598 for Control [High], 204.571 ± 3.599 for NeuroD1 [Low], 205.889 ± 4.885 for NeuroD1 [High]. The average force applied to spinal cords for subacute treatment were 206.556 ± 5.175 for SCI Only, 206.818 ± 2.750 for Control [Low], 203.625 ± 4.033 for Control [High], 204.727 ± 1.954 for NeuroD1 [Low], and 207.000 ± 4.522 for NeuroD1 [High]. All data shown are presented as mean ± standard deviation. There was no significant difference between groups as evaluated by *one-way ANOVA followed by a Tukey's post hoc test.*

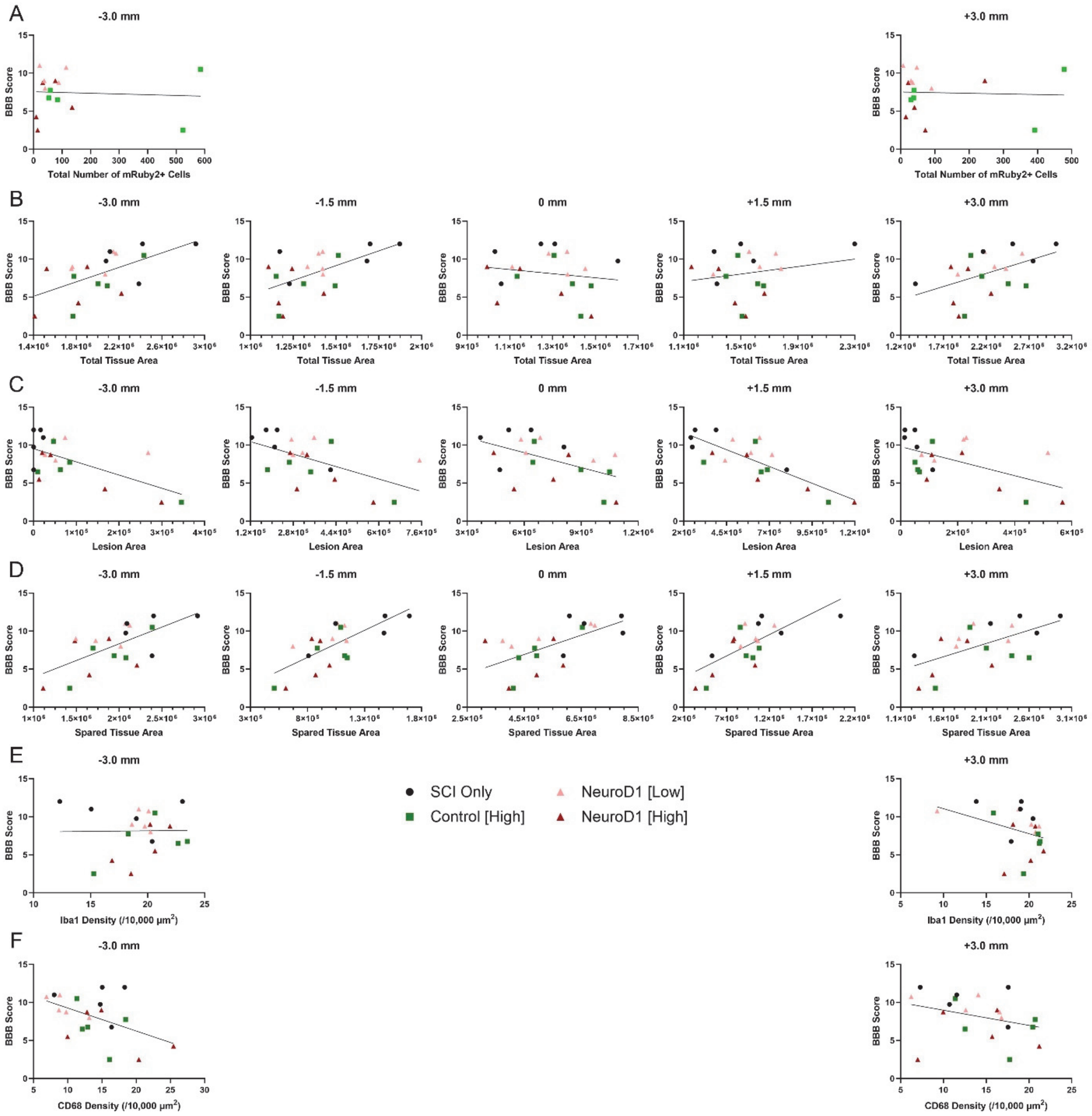

Figure S2. Correlations with BBB Scores at Defined Distances from Lesion Site. Pearson correlations between BBB locomotor score at 6 WPC and **(A)** total number of mRuby2+ cells, **(B)** total spinal cord tissue area, **(C)** lesion area, **(D)** spared tissue area, **(E)** Iba1 density, and **(F)** CD68 density across all acute treatment groups: SCI Only, Control [High], NeuroD1 [Low], and NeuroD1 [High]. Correlations were made at select distances from the injury center: −3.0 mm, −1.5 mm, 0 mm (injury center), +1.5 mm, and +3.0 mm. Negative distances are rostral to the injury site and positive distances are caudal. Linear regressions were performed on the datasets, with Pearson correlation coefficients and p-values recorded in the associated **Table 1.**

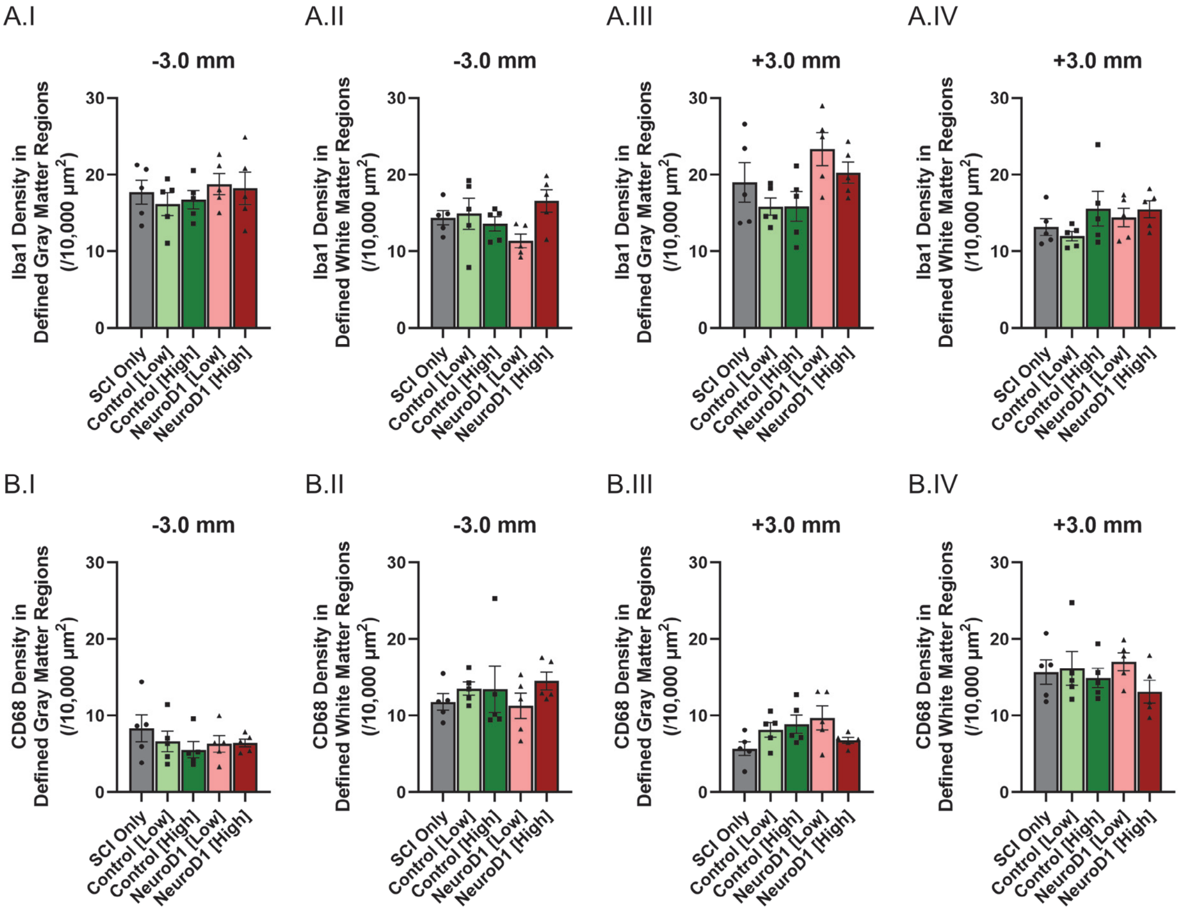

Figure S3. Effect of subacute NeuroD1 treatment on microglia/macrophage density. Sections of spinal cord, located at −3.0 mm and +3.0 mm, were stained with antibodies for CD68 and Iba1. The density of (**A**) CD68+ cells and (**B**) Iba1+ cells was quantified within defined gray and white matter regions of the spinal cord sections. All data shown are presented as mean ± standard error.
